# Ecological Momentary Assessment and Smartphone Application Intervention in Adolescents with Substance Use and Comorbid Severe Psychiatric Disorders: Study Protocol

**DOI:** 10.3389/fpsyt.2016.00157

**Published:** 2016-09-20

**Authors:** Xavier Benarous, Yves Edel, Angèle Consoli, Julie Brunelle, Jean-François Etter, David Cohen, Yasser Khazaal

**Affiliations:** ^1^Department of Child and Adolescent Psychiatry, Hôpital Pitié-Salpêtrière, Paris, France; ^2^Department of Addiction, Hôpital Pitié-Salpêtrière, Paris, France; ^3^Institute of Global Health, University of Geneva, Geneva, Switzerland; ^4^UMR 7222, Institute of Intelligent Systems and Robotics, Université Pierre et Marie Curie, Paris, France; ^5^Faculty of Medicine, Geneva University Hospitals, University of Geneva, Geneva, Switzerland; ^6^Geneva University Hospitals, Geneva, Switzerland

**Keywords:** substance use disorder, cannabis addiction, prevention, adolescents, ecological momentary assessment, ecological momentary intervention, mHealth app, smartphone app

## Abstract

**Context:**

Substance use disorders (SUDs) are highly prevalent among inpatient adolescents with psychiatric disorders. In this population, substance use and other psychiatric outcomes can reinforce one another. Despite the need for integrated interventions in youths with dual diagnoses, few specific instruments are available. App-based technologies have shown promising results to help reduce substance use in adolescents, but their applicability in youths with associated severe psychiatric disorders is poorly documented. We aim to evaluate the feasibility of an ecological momentary assessment (EMA) intervention for all substance users, and of a smartphone application for cannabis users (Stop-Cannabis), for outpatient treatment after hospital discharge.

**Methods and analysis:**

All inpatient adolescents with psychiatric disorders hospitalized between 2016 and 2018 in a university hospital will be systematically screened for SUD and, if positive, will be assessed by an independent specialist addiction team. Participants with confirmed SUDs will be invited and helped to download an EMA app and, if required, the Stop-Cannabis app, the week preceding hospital discharge. Information about the acceptability and use of both apps and the validity of EMA data in comparison to clinical assessments will be assessed after 6 months and 1 year.

**Discussion:**

This research has been designed to raise specific issues for consideration regarding the sequence between substance use, contextual factors, and other psychiatric symptoms among adolescents with comorbid severe psychiatric disorders. A better understanding of the mechanisms involved will inform the development of integrated treatment for dual disorders at that age.

**Ethics and dissemination:**

The study has already been approved and granted. Dissemination will include presentations at international congresses as well as publications in peer-reviewed journals.

**Trial registration:**

European Clinical Trials Database: Number 2016-001999-30.

## Introduction

### Background

#### Substance Use Disorders and Psychiatric Disorders in Adolescents

Psychiatric comorbidity in adolescents who abuse substances is the rule rather than the exception, with 61–88% having a dual diagnosis (1, 2). The coexistence of both disorders is associated with important disruptions in a range of functional domains, including academic attainment, social and family relationships (2, 3), and accidents (4). In addition, initiation of substance use at a younger age (5) and a faster relapse after treatment (6) are reported in youths with preexisting mental health problems. In turn, the natural course of psychiatric disorders may be affected by substance use, either due to negative psychosocial consequences associated with substance involvement (7) and/or to the direct effect of the substance on the central nervous system (8). In youths with mood or psychotic disorders, for example, daily use of cannabis is associated with a more severe clinical presentation, impaired cognitive and interpersonal abilities, and poorer responses to pharmacological treatments (9). The development of integrative treatment for substance use disorders (SUDs) and psychiatric disorders is therefore a priority (10, 11).

Among adolescents hospitalized for a psychiatric problem, 17–50% meet criteria for one or more SUDs (12–17). In a previous study of adolescents hospitalized in three psychiatric units in France, most subjects met criteria for a non-nicotine SUD: around 70% for cannabis, 60% for alcohol, and 20% for other substances (18). Unsurprisingly, those with dual disorders exhibited more severe clinical characteristics (e.g., more suicide attempts) and a higher level of functional impairment (e.g., more school absenteeism) than patients without SUDs. They also received more diagnoses of axis 2 comorbidities and were exposed to more severe psychological contexts (e.g., maltreatment or loss of a first relative) (18). Given the high prevalence of SUDs among adolescents who are hospitalized, and the poor clinical outcomes due to the negative interplay between both disorders, specific preventive interventions for addiction should be developed and evaluated in this population.

#### Addiction Preventive Interventions in Inpatient Adolescents with Psychiatric Disorders

It is well established that treatments for SUDs and psychiatric disorders are less effective when provided separately (1, 6). Although the efficacy of integrated approaches for the management of dual disorders has been demonstrated in adolescents (10, 11), there is some concern that – in practice – the so-called “integrated approach” often consists of the addition of evidence-based treatments for both problems, with too few specific dual diagnostic or therapeutic tools. For example, in order to choose the optimal treatment, the sequence between substance use and other psychiatric symptoms in patients’ daily life should be explored, but we are not aware of any tool that would measure this pattern. Furthermore, treatment compliance is a key prognostic factor in addiction. For most teenagers, SUDs are commonly under-recognized as a health issue (19). The development of tools that promote better recognition of patients’ problems with substances, and enhance behavior change, would therefore be worthwhile. Digital approaches may be a promising avenue for health-related interventions among adolescents given their propensity to use digital technologies (20–24).

#### Digital Approaches

The uptake of smartphones in the general population has encouraged the development of apps used to facilitate real-time assessment of the patient in his/her natural environment [Ecological Momentary Assessment (EMA)] and allowed delivery of smartphone app interventions in this context.

Ecological momentary assessment consists of using computers or other devices to collect self-assessments repeatedly in real-time, in various situations of the participant’s daily life, either for research purposes or to provide participants with timely feedback and advice (25). Different terms have been used for this methodology since the 1980s, including “ambulatory assessment,” “experience sampling method,” or “real-time data capture.” Even though the terms differ, these approaches have the data collection in common (e.g., symptoms, behaviors, physiological processes, or daily life circumstances) while the participant undergoes normal activities. Therefore, this allows the interplay between contextual stressors, psychological distress, and maladaptive behaviors to be modeled (26). Compared to traditional measures using paper–pencil questionnaires, EMA is regarded as having many benefits for addiction research, including decreased recall bias, decreased contextual bias (25, 27), and increased validity to model the temporal sequence of events between risky situations, craving, and substance use (28, 29). Although such tools have been available for over 30 years, the emergence of smartphone applications as a platform for EMA greatly improved the acceptability and usability of this technology (30). In the last decade, EMA systems have been developed for adolescents with addictions (31) and applied to the assessment of psychiatric symptoms in outpatients with internalizing/externalizing disorders (32–36). In contrast with adults [e.g., Ref. (37)], EMA has not yet been assessed, to our knowledge, for studying the relationship between psychiatric symptoms and patterns of substance use in adolescents. In addition, the feasibility of using EMA in adolescents with severe psychiatric symptoms remains understudied.

The smartphone app Stop-Cannabis was developed in 2013 in Geneva, Switzerland with financial support from the local Health Department.^1^ The app aims to help users reduce or stop their cannabis use and prevent relapse. Stop-Cannabis consists of several modules based on cognitive behavioral therapy strategies, motivational interviewing, and relapse prevention programs. Particular effort has been made to include the principles of self-determination (autonomy, competence, and relatedness) (38) into the app and promote its customization (39). As in other studies (40–42), participant interaction and rewards support the playful aspect of the intervention. Personalization of the users’ goals and the app main screen may further contribute to increase adoption by user (43). The contents of the application are summarized below [and described in detail in Ref. (39)]. Since February 2013, the Stop-Cannabis app [available on Google Play (Android) and (iOS) App Store] has been downloaded more than 13,000 times, and the app is currently used by 1,000 people every month. The app was reported to have a good level of acceptability among community users in an online satisfaction survey (39).

This study is part of a general collaborative research program between the child and adolescent psychiatric department team and the addiction unit. The purpose of this wider program is to inform the difference in the trajectories of inpatients adolescents with SUDs compared to other and to help developing a better clinical practice (i.e., early detection, clinical assessment, and therapeutics).

### Objectives

The primary objective of our pilot study is to evaluate the feasibility and acceptability of two different digital tools (an EMA tool and Stop-Cannabis) based on smartphone apps in adolescents with dual disorders during a 1-year follow-up period after hospital discharge. We aim to determine the feasibility of the EMA, adherence to recommendations, and the quality of data collected using an EMA digital assessment tool. In addition, we aim to evaluate the feasibility, acceptability, and use of the Stop-Cannabis app, a digital intervention tool, during outpatient aftercare. The authors expect to obtain from this pilot study data on the feasibility and acceptability of two kinds of digital tools (one related to assessment and the other one to an intervention) possibly useful for adolescents with SUDs and comorbid severe psychiatric disorders.

Secondary objectives are (1) to estimate the prevalence of SUDs in adolescents with psychiatric disorders hospitalized in the Department of Child and Adolescent Psychiatry at the Pitié-Salpêtrière University Hospital, Paris, France (this department provides one-third of all child and adolescent inpatient beds in the Paris area); (2) to describe the clinical and psychosocial characteristics of substance users compared with non-users; (3) to determine the risk factors for the onset of SUDs during follow-up in participants without dual diagnoses at baseline; (4) to determine the risk factors for the persistence of non-cannabis SUDs during follow-up; (5) to document the longitudinal interplay between substance use and psychopathology in adolescents with dual diagnoses at baseline; and (6) to examine the association between the use of the Stop-Cannabis app and the patterns of substance use at 1-year follow-up in naturalistic settings to inform the potentiality for future research.

## Methods and Analysis

### Selection of Participants

#### Inclusion and Exclusion Criteria

Participants will be adolescents (11–18 years old) who are hospitalized in the Department of Child and Adolescent Psychiatry at the Pitié-Salpêtrière University Hospital between September 2016 and September 2018. The participants will be assessed at baseline. According to the presence or absence of comorbid SUDs, they will be included in either the SUDs group or the control group. The SUDs group will receive the mobile apps, whereas the control group will not. Exclusive tobacco consumption is not considered to be a criterion for inclusion in the SUDs group. Inclusion to the non-SUD control group will be made according to a match with adolescents from the SUDs group (for age, gender, inpatient unit, and time of admission). Exclusion criteria include non-French speaking, those without a personal mobile phone, lack of informed consent from parents or from the adolescents, and any mental or physical problems that interfere with participation in the study (e.g., autism with very low communication abilities, severe to profound intellectual disability, severe motor disability).

#### Sample Size

A conservative approach was adopted for this feasibility study, which emphasizes descriptive and qualitative feedback. Consequently, based on the recommendations for pilot studies (44), we plan to include 80 participants (40 cases, 40 controls) over a 2-year inclusion period. As there are around 280 inpatient admissions per year in the Department of Child and Adolescent Psychiatry, i.e., *n* = 560 potential participants contacted at baseline during the 2 years, this will represent a 14% inclusion rate. We predict that the prevalence of SUDs will be similar to that previously reported (18). A good level of compliance with EMA and Stop-Cannabis is expected, consistent with previous studies that reported high levels of adherence (up to 80%) to similar tools in adolescents with comorbid psychiatric disorders (31, 45), and in adults with associated severe psychiatric disorders (32, 46). An acceptance rate of 80% is expected in the SUD group and 50% for the control group. A 50% retention rate at 1-year follow-up is anticipated based on previous longitudinal studies among adolescents (47). In case of a lower enrollment rate, we will either continue to enroll participants during an additional year (2019) in order to reach the intended sample size or use incentives (e.g., free participation in leisure and sport activities in groups) to encourage participant enrollment.

### EMA and Stop-Cannabis App Tools

#### Ecological Momentary Assessment

The EMA was designed as an app for mobile phones. This application allows several daily self-assessments (e.g., five times a day for 2 weeks at different times of the study/monthly). Data are collected in response to a signal from the EMA app that occurs at various times of the day. The app has been programed to send signals (i) a fixed number of times per day at predetermined times (e.g., at bedtime) and (ii) at random (with a balance between week, weekends, and holidays, with different time intervals in daytime). Participants are also asked to complete self-assessment immediately following substance use.

The same questions are presented regardless of assessment type. There are seven questions in total:

- Information relating to substance use:
  ○ Daily use of each substance in list.
  ○ Level of withdrawal symptoms during participants’ most recent period of abstinence from 0 (not at all) to 5 (severe).
  ○ Level of craving from 0 (no urge) to 5 (extreme urge).
- Information relating to psychopathology:
  ○ Level of negative affect rate using a brief version of the Positive and Negative momentary Affect Scale (PANAS) (48) adapted for EMA (32). Participants provide ratings of the extent to which they felt each emotion on a 1–5 point Likert-type scale.
  ○ Main psychiatric symptoms.
- Information relating to environment:
  ○ Stressful events.
  ○ Activities: physical activity, planned activity, lone activity, social activity with peers, family activity, and online activity.

Participants will be trained on EMA use during the week prior to hospital discharge. They will be asked to respond to any signals within 1 h if possible. Consistent with other EMA protocols, participants will complete 2 days of practice data (not used for analyses), after which they will receive feedback on adequate use and compliance. Participants will then be invited to complete EMA assessments for 2 weeks, as this timeframe appears sufficient to monitor substance use (45). To limit attrition, participants will be paid 30€ for completing the baseline assessment and 10€ for each week of complete EMA data, up to a maximum possible payment of 60€ over 1 year.

#### The Stop-Cannabis App

**Figure 1:**
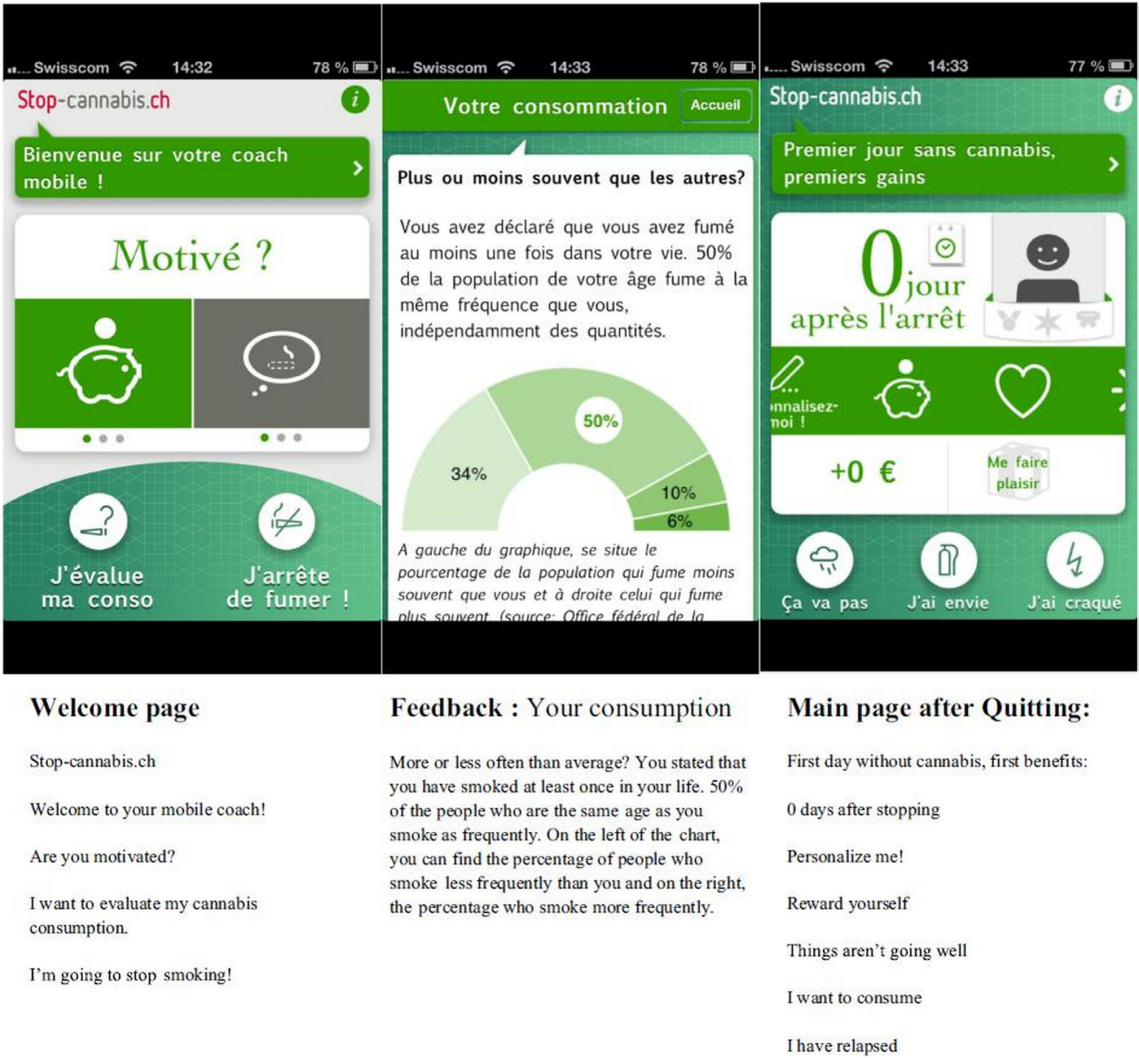
**Ecological momentary intervention “Stop-Cannabis.”** *Notes*: screenshots of the welcome page, the Feedback page, and the main screen with the benefits of change. Adapted from: Monney et al. (39).

Launched in 2013, Stop-Cannabis is available free on iOS and Android. It has been continuously updated and improved over the past 3 years, in response to users’ suggestions. When people use the app for the first time, they are asked to document their objectives and choose a quitting date. The app has several modules. First, it allows users to assess their cannabis usage profile (including motivations for use and severity of cannabis use) *via* different assessments (49). Users then receive an individually tailored feedback message that includes links to online psychoeducation and complementary resources. Second, motivational messages are regularly delivered using push-notification technology at different stages of the quitting process, on the basis of updated information regarding current substance use, the selected quitting date, and exposure to situations associated with a high risk of relapse documented by high levels of craving or irritability. Third, the app includes messages on how to cope with craving symptoms or emotional difficulties to enhance participants’ insight into contextual factors for substance use. Fourth, positive reinforcers, such as the number of days since quitting the substance and the amount of money saved, are displayed on the main screen and can be personalized (Figure 1). Fifth, a discussion forum called “The Tribe,” moderated by a psychologist, is available for all users to encourage discussion, mutual support, and information sharing. Sixth, the app includes direct access to a website^2^ for additional information about substance use and online motivational interviewing training.

The app will automatically monitor the number of times each participant opens the app and records the number of times participants looked at or used each element. A Unique Device Identifier (UDI) is used to connect the participants’ device to the study database and will be replaced in the data set with a random ID for anonymity. After study completion, the UDIs will be automatically deleted from users’ devices. The app will be available for participants in the intervention group for 12 months. After this period, the app will automatically stop data collection. However, participants are free to use the app for as long as they want.

### Design

#### Screening

**Figure 2:**
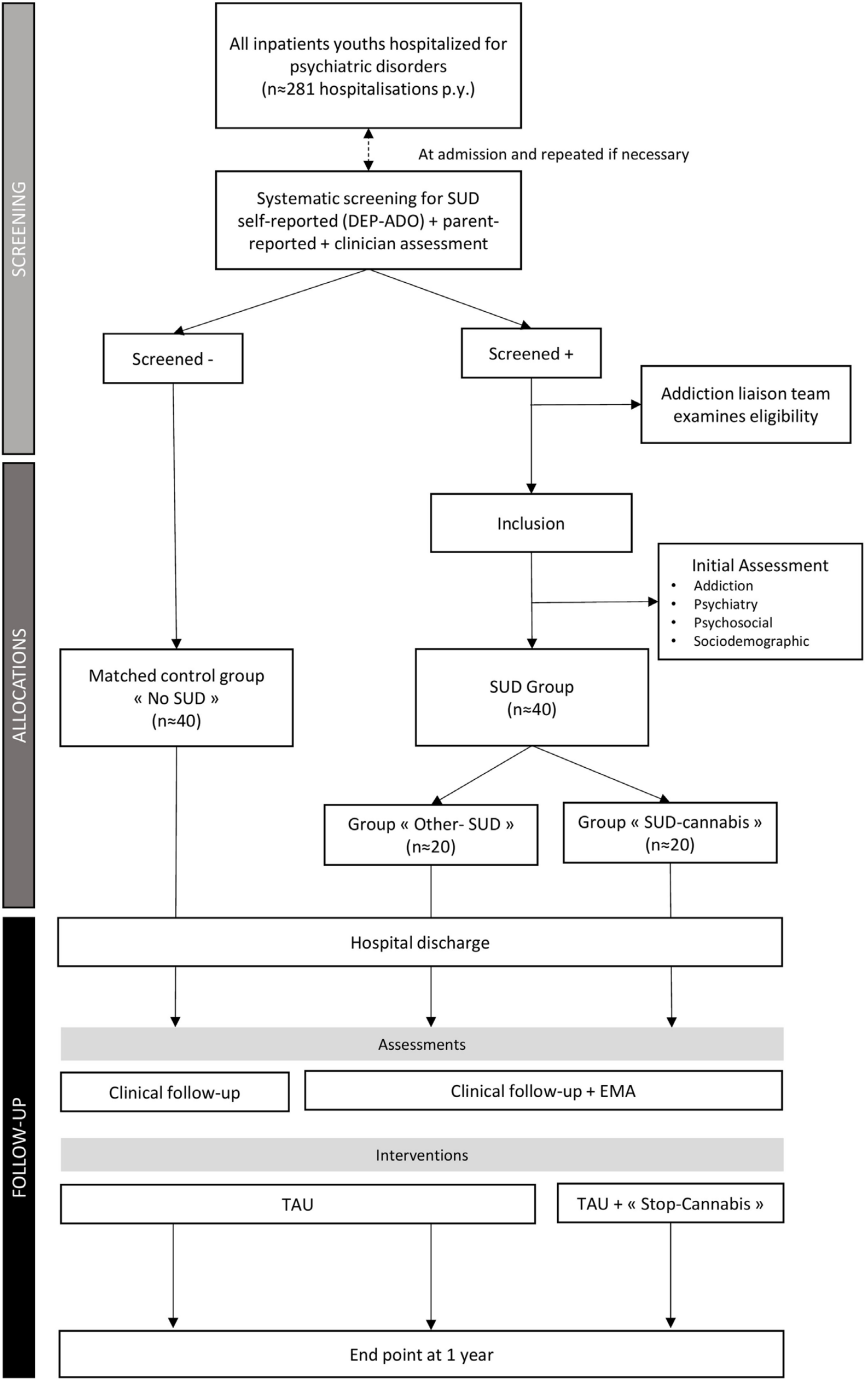
**Flow-chart of the study**. *Notes*: SUD, substance use disorders; DEP-ADO, the questionnaire used for participants’ screening, is detailed in Section “Screening”; EMA, ecological momentary assessment; TAU, treatment as usual.

The study flowchart is shown in Figure 2. Each patient admitted to the Department of Child and Adolescent Psychiatry at the Pitié-Salpêtrière University Hospital during the study recruitment phase will be systematically screened for SUDs and eligibility at admission. The DEP-ADO questionnaire (50) will be used in the study to document substance use in the previous 12 months. The complete questionnaire is available free online^3^. The screening question will be “During the last twelve months, how often have you [has X] used one of the following substances: alcohol, cannabis, cocaine, inhalant/solvent, stimulant, hallucinogen, or heroin” (examples and trivial names are provided for each substance). For each substance, participants and their families will have the options of answering: “Never,” “Occasionally,” “Once per month,” “Once or twice per week,” “More than three times per week,” or “Daily.” The clinician version of the DEP-ADO will be completed by the clinician involved in usual care after an interview with the adolescent and his/her family. The self-report version will be completed by the adolescent, with assistance from a specially trained member of the paramedical staff. Assessments will be carried out within 3 days following admission and repeated after 1 week as an inpatient, and again if necessary (especially if the clinical condition is not compatible with self-assessment).

Urinary drug tests will be performed on all adolescents hospitalized in the Department of Child and Adolescent Psychiatry during the study recruitment phase. The tests screen for the presence of cannabis, cocaine, opiates, and amphetamines. In the interest of having as broad as possible a representation of the target population, we will include as “screen positive” those who answer at least “occasionally” to use of cocaine, inhalant/solvent, stimulant, hallucinogen, or heroin, and/or at least monthly use of cannabis or alcohol, and/or when their urine test indicates the presence of one of these substances.

#### Assessment to Determine Eligibility

All youths screened positively for substance use will be reported to the hospital’s liaison addiction unit (ECIMUD, a French acronym for *coordination and medical care team for addictive disorders)* for proper diagnosis and care. Systematic evaluation of the substance use and habits will be conducted *via* a semi-structured interview, which allows for scoring the RECAP index, the clinician version of the DEP-ADO, and the Cannabis Use Screening Test (CAST), a self-reported scale that focus on use of cannabis in the previous 6 months (51). Final diagnoses of SUDs will be made by one of the ECIMUD senior psychiatrists using DSM-5 criteria (52).

#### Initial Clinical Assessment and Follow-Up

Participants (i.e., adolescents with SUDs and control subjects) will be approached by a researcher involved in the project (either a clinician specialized in child psychiatry or a master’s level psychologist) who will explain the study and obtain written informed consent from parents or guardian and assent from the adolescent. Participants will be assessed in the week prior to hospital discharge for sociodemographic data, psychosocial information, psychopathology, and substance use (see Variables and Instruments). All youths will be assessed in face-to-face interviews by a specially trained master’s level psychologist at 6-month and 1-year follow-up.

### Data Analysis

#### Variables and Instruments

Table 1 lists the questionnaires and other assessments that will be used at baseline and at follow-up. The reading level of the questionnaires is grade 6. The questionnaires we plan to use to measure addiction are described below. A combination of self- and clinician-report measures with different time scales (i.e., DEP-ADO, 12 months; CAST, 6 months; RECAP, 1 month) will be used to examine whether EMA data are consistent with information collected in the traditional way. The second type of measures relate to psychopathology with the aim of examining the interplay between substance use and psychiatric disorders.

**Table 1:** **Variables examined in the study**.

Variables	Administration	Initial assessment	Month 6	1 year
**Substance use**				
DEP-ADO	Clinician-report self-report	X		X
RECAP Index	Clinician-report	X	X	X
CAST	Self-report	X	X	X
ICSU	Self-report	X		X
Misuse of caffeinated sodas	Self-report	X		X
Urinary screening test	Biological test	X		
**Psychopathology**				
K-SADS-PL	Semi-structured interview	X		X
Ado DIB	Self-report	X		X
ARI	Parent-report	X	X	X
	Self-report			
UPPS-P	Self-report	X	X	X
C-GAF	Self-report	X	X	X
**Other variables**				
Age, gender, academic status, socioeconomic status	Parent-report	X		
ACE	Self-report	X		
Satisfaction questionnaire	Self-report		X	X
CSSRI-EU	Clinician-report	X	X	X

*DEP-ADO, RECAP Index, CAST, Cannabis Use Screening Test; ICSU, Compulsive Internet Use Scale; K-SADS-PL, Kiddie-SADS-Present and Lifetime Version; Ado DIB, Adolescent Diagnostic Interview for Borderline Patients; ARI, Affective Reactivity Index; UPPS-P, Impulsive Behavior Scale; C-GAF, Children’s Global Assessment of Functioning; ACE, Adverse Childhood Experiences Questionnaire; CSSRI-EU, Client Sociodemographic and Service Receipt Inventory-European Union*.

##### Substance Use

- The RECAP index is a clinician-reported questionnaire systematically used for standardized collection of information regarding substance use during the last month in outpatient addiction centers in France, recommended by the EMCDDA (European Monitoring Centre for Drugs and Drug Addiction) (available at http://www.ofdt.fr/enquetes-et-dispositifs/recap/presentation/).
- The CAST is a 6-item self-reported scale that focuses on the use of cannabis in the last 6 months, validated in French (51).
- The Compulsive Internet Use Scale (ICSU) is a 14-item self-reported scale that focuses on compulsive internet use (53), validated in French (54).
- The pattern of use of caffeinated sodas will be assessed using a clinician-reported scale previously used in a French outpatient non-clinical sample (55); items are based on Goodman’s criteria for addiction (56).

##### Psychopathology

- The Kiddie-SADS-Present and Lifetime Version (K-SADS-PL) is a semi-structured interview used to assess psychiatric disorders (57).
- The Affective Reactivity Index (ARI) is a 7-item clinician-reported scale that focuses on distinct domains of irritability (i.e., tonic and phasic), validated in English (58).
- The Impulsive Behavior Scale (UPPS-P) is a 20-item self-reported scale that focuses on distinct domains of impulsivity (i.e., sensitivity to positive and negative emotions, lack of perseverance, lack of premeditation, and sensation-seeking), validated in French (59).
- The Adolescent Diagnostic Interview for Borderline Patients (Ado DIB) is a self-reported questionnaire that assesses the diagnostic criteria for borderline personality disorder. Translation and validation in French are provided by Guilé and colleagues (60).
- Overall functioning will be measured using the Children’s Global Assessment of Functioning (C-GAF), one of the most widely used measures of the overall severity of adolescent disturbance (61).

##### Other Measures

- Sociodemographic data, including age, gender, academic status, and socioeconomic status.
- Adverse life events, collected using the Adverse Childhood Experiences Questionnaire (ACE), a 10-item self-reported scale that explores the presence of abuse and severe neglect during childhood, validated in English (62). Other psychosocial variables include living arrangements (classified into four groups: stable family, unstable family, stable institutional care, unstable institutional care), school absenteeism (partial or complete), orphan or adopted child, first-degree loss, family dysfunction (e.g., parental conflict, parental separation, divorce), and educational support.
- The Client Sociodemographic and Service Receipt Inventory-European Union (CSSRI-EU), a questionnaire completed by the clinician that details treatment received (including pharmacological treatments) and the type of care (such as the number of hospitalizations, outpatient services, emergency admission) (63).
- Satisfaction questionnaires for EMA and Stop-Cannabis will be completed at 6 months and at end-point (1 year).

#### Outcomes

Information will be collected from both EMA data and from questionnaires administered during hospitalization and follow-up.

##### Primary Outcomes

The first parameter is whether the level of adherence and the acceptability of the EMA and the Stop-Cannabis app are satisfactory in this sample. Acceptability will be assessed by measuring the dropout rate after 6 and 12 months, and usability and satisfaction *via* questionnaire in all participants and *via* face-to-face interviews with a subgroup of 10 participants. Compliance will be assessed *via* mean percentage of random prompts and predetermined assessments completed per participant. The second parameter is whether information collected using EMA *via* a mobile app is valid and reliable. Information obtained by EMA tools using the mobile app will be compared to those collected by traditional paper–pencil and clinical interview methods during the initial and follow-up assessments using correlations analysis.

##### Secondary Outcomes

(1) The prevalence of SUDs among adolescent inpatients will be assessed.
(2) The clinical and psychosocial characteristics of participants with and without SUDs at baseline, including history of adverse childhood experiences, comorbid psychiatric diagnoses, and the level of functional impairment, will be compared.
(3) The risk of developing SUDs among non-SUD adolescents during a 1-year follow-up period following hospital discharge will be investigated.
(4) The proportion of inpatient adolescents with non-cannabis substance use at baseline whose SUDs persist at 1 year will be estimated.
(5) We will explore how psychiatric symptoms could affect the pattern of substance use among SUDs participants.
(6) We will assess whether specific patterns of Stop-Cannabis app use is associated with substance use outcomes at 1-year follow-up.

#### Statistical Methods

##### Primary Outcomes

Pearson correlations will be used to determine the strength of associations between daily substance use assessed by either EMA, or self-report questionnaire (e.g., CAST), or by clinician-report questionnaire (e.g., RECAP) during the clinical assessment at 6 months. Clinical reliability of the EMA will be endorsed if the correlations are statistically significantly higher than 0.70. Further analyses would be performed to estimate different aspects of reliability of the data collected in line with Perrez et al. (64). The severity of SUDs at baseline should predict the severity of substance use and the level of functional impairment at 1 year at least to comparable levels while using EMA data at baseline compared with traditional methods. Two simple linear regression models will be performed. For both the models, the dependent variable will be the severity of SUDs at follow-up; in one model, the independent variable will be the severity of SUDs based on EMA data (Model A), whereas in the other model, the variable will be severity of SUDs based on self- and clinician-report (Model B). The estimate of the independent variable is expected not to be lower in the Model A than in the Model B.

The dropout rate is expected to be less than 40% at 6 months and less than 50% at 12 months for both the EMA and traditional method, with no significant difference between methods. The results of the satisfaction survey will be estimated by analyzing the percentage of answers and the differences among the different categories using Chi-square analysis.

##### Secondary Outcomes

(1) The number of inpatients with SUDs will be compared with the total number of adolescents admitted to the Department during the inclusion period.
(2) Descriptive analyses of clinical and psychosocial variables will be conducted. Chi-square will be used to compare categorical variables, and Student’s *t*-test for continuous variables. A difference of *p* < 0.05 will be considered statistically significant.
(3) Univariate logistic regression analyses will be performed to determine whether clinical features (as independent variables) predict the onset of SUDs at 1 year (as dependent variable) in the non-SUD control group and persistence or cessation in the SUD group. Possible mediators of this relation will be examined using the four-step approach proposed by Baron and Kenny (65), followed by a Sobel–Goodman test. In particular, we will test the mediation effect of the baseline impulsivity score, the baseline level of sensation-seeking (subscales of the UPPS-P score), and the baseline level of mood lability (ARI total score) on the relationship between psychopathology and SUDs.
(4) Comparable analyses would be conducted to determine the clinical and psychosocial risk factors for the persistence of SUDs among those with non-cannabis substance use at baseline.
(5) Specific analyses based on mixed-effect models (a random effect for participants and fixed effects for time) will be performed to examine the interplay between the pattern of substance use, psychiatric symptoms, and contextual stressors for treating EMA data [as in Ref. (45, 66)]. In line with previous recommendations, data will be studied at the daily level (with average ratings), at the concurrent momentary level, and at the prospective level using both linear and non-linear modeling (32). These analyses will be performed and presented separately between those with cannabis use disorder and the other participants to prevent any interference due to the use of the Stop-Cannabis app.
(6) Correlational analyses will be carried out to examine the association between the level of app use (EMA or Stop-Cannabis) (i.e., mean percentage of random prompts, mean percentage of days assessment completed) and participant’s clinical outcome at follow-up (i.e., frequency of substance use and level of functional impairment at 1 year).

#### Calendar

March 2016: training the medical team to use the screening tools (i.e., DEP-ADO).
December 2016: start of participant inclusion (t0).
December 2017: end of data collection for participants enrolled at t0.
November 2018: end of inclusion.
December 2018 to January 2019: start of analysis of baseline data, i.e., prevalence of SUDs.
November 2019: end of data collection for final participants included.
December 2019 to January 2020: analysis of final data.
February 2020: preparation of an abstract for submission to psychiatric congress.
April 2020: writing one to three articles for psychiatric and addiction journals.

## Discussion

### Anticipated Results

#### Primary Objectives

A degree of correlation higher than 0.70 is expected between EMA data and data collected using other methods (particularly frequency of use), in line with prior studies (46). EMA should lead to predictions regarding the course of SUDs over the 1-year follow-up (e.g., in term of persistence, severity, and functional impairment) at least as good as other methods; that would be of interest for the clinicians involved in usual care. A high level of compliance with EMA is expected, despite the relative severity of the study sample. Based on previous studies in adolescents (45, 67), participants are expected to complete a mean of more than 70% of random signals, 50% of end-of-day assessments, and 40% of both random and end-of-day assessments. Indeed, there is good evidence that EMA can be used by patients with severe psychiatric disorders (e.g., schizophrenia) or cognitive difficulties (32, 46).

#### Secondary Objectives

This study will measure the prevalence and the characteristics associated with the presence of SUDs in a sample of adolescents hospitalized in a psychiatric setting. Findings are expected to be in a similar range as those previously reported by Daudin et al. (18). We will then examine the possible relationship between SUDs and specific psychosocial and clinical features of inpatient adolescents. We expect this study to confirm that both internalized and externalized disorders are significantly associated with SUDs and that the presence of SUDs is associated with a higher level of impulsivity, mood dysregulation/borderline traits, and sensation-seeking traits (68–70). We predict that a bidirectional positive relationship between the level of substance use and the severity of other psychiatric symptoms will be noted at a daily level, and – if confirmed – that this association would be strengthened in a context of interpersonal relationship (with family and with peers) compared to other situations. A significant negative association between the level of the Stop-Cannabis app use and the participant’s clinical outcome at follow-up is expected. Such additional exploratory analysis would provide preliminary information about the possible benefit of the app in naturalistic settings.

### Limitations

Several limitations may preclude interpretation of our findings. As no control group for the Stop-Cannabis app is defined (i.e., subjects with cannabis use disorders without the intervention), the effectiveness of the Stop-Cannabis app to reduce or quit cannabis cannot be directly examined in our study. Furthermore, one other limitation is related to the lack of EMA assessments for the control group. The groups cannot be then compared with the EMA data. The authors choose to not use EMA assessment for the control group in order to avoid possible impact of the assessments on the behaviors [for a discussion about the reactivity effect of EMA, see Shiffman (25)]. The groups will receive, however at different time, a number of clinical assessments useful for the planned comparisons. As our study participants will be recruited during hospitalization in a university teaching hospital, the sample is likely to comprise youths with severe or resistant clinical profiles, which could limit the generalizability of our findings. In addition, the study design requires that only participants who own a smartphone are included, which is a risk for selection bias. Attrition biases may result from high dropout rates and low use of the app, but incentives and reminder phone calls will be employed to improve adherence. However, if important differences in baseline characteristics between adherent and non-adherent participants are detected, this will be further controlled by regression analyses. Finally, no firm conclusion about the benefit of the Stop-Cannabis application can be drawn from this study with regards to the lack of controlled randomized group.

### Clinical and Research Implications

**Figure 3:**
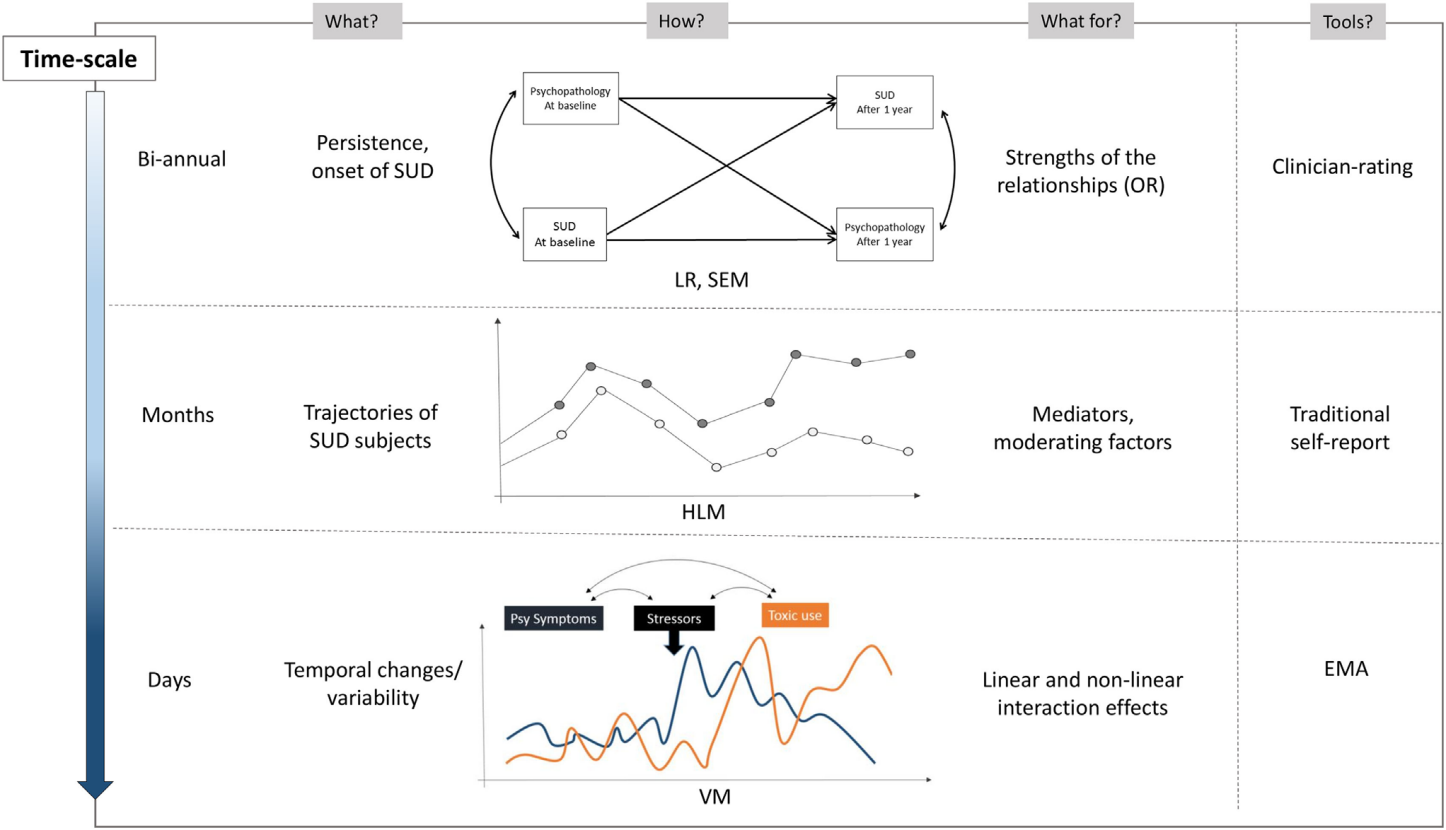
**Ecological momentary assessment and other methods to study the relationship between psychopathology and substance use during follow-up**. *Notes*: OR, odds ratio; LR, logistic regression; SEM, structural equation modeling; HLM, hierarchical linear modeling; VM, variance modeling. EMA collects information regarding substance use, craving sensation, emotional state, coping strategies, nature of stressors, withdrawal symptoms, activity, environmental context, and social interaction.

The utility of app tools for adolescents with dual disorders would be of clinical interest, considering the need for evidence-based treatments and interventions in this population (71). Longitudinal data and mediation analyses would provide preliminary data on the mechanisms involved in the relationship between dual disorders (Figure 3). A better understanding of this mechanism is essential to focus on specific subpopulations in pursuit of more integrated treatment and support for their mental health and addiction problems. The originality of this study stems from the combination of tool-based measures with distinct temporal scale: standardized semi-structured interviews at baseline and end-point, self-reported non-ecological data obtained every 6 months, and self-measurement in the individual’s natural environment using EMA tools.

Ecological momentary assessment data provide insights on the sequence between substance use, contextual factors, and psychiatric symptoms that could not have been obtained using more traditional self-report measures. This is particularly important for adolescents where psychopathology may be more fluctuating and context-dependent than in adults. In contrast with previous EMA data research projects conducted in children and adolescents, our sample encompasses a large range of psychiatric disorders with a more severe profile. Finally, in line with other studies (72), the insights obtained from EMA may not only serve researchers and clinicians but might also benefit patients directly as patients become increasingly aware of their symptoms.

Finally, this research will provide information that ultimately helps determine whether a randomized controlled trial could be conducted in the future to test the benefit of a digital intervention based on the Stop-Cannabis app in adolescents with severe comorbid psychiatric disorders.

## Ethics and Dissemination

### Informed Consent and Institutional Review Boards

Regarding patient safety, all participants will receive detailed information about local help in case of crisis, emergency, or suicidal ideation. Participants will also be informed about, and trained how to use, the apps and told what can be expected from their use. They will also be advised to consult their doctor and share concerns with their family and relatives according to their needs. Participants will be instructed to not use the apps when it is inconvenient (e.g., in class) or unsafe (e.g., on bikes).

Informed consent will be obtained from the adolescent and his/her family or legal representatives after they have received information about the study objectives and procedures. Agreement has already been provided by the scientific board of the Institute of Research in Public Health; however, data collection will only start when the protocol will be approved by the Ethics Committee (EC) of the Pitié-Salpêtrière Hospital. In the case of protocol changes, an amendment will be submitted to the concerning EC. The project has already received financial support from French institutions, i.e., la Direction General de la Santé (DGS), la Caisse Nationale de l’Assurance Maladie des Travailleurs Salariés (CNAMTS), la Mission interministérielle de lutte contre les drogues et les conduites addictives (MILDECA), and l’Observatoire national des Jeux (ODJ) on the basis of an open invitation to tender from the IreSP in 2015 (reference “IReSP-15-Prevention-11”).

The study protocol has been recorded on the European Clinical Trials Database (EudraCT Number 2016-001999-30).

### Confidentiality

In order to ensure patient confidentiality during the study and transmission of personal data, a security protocol using a digital identification number will be employed.

The collected data will be only accessible to the principal investigator and study staff as well as the monitors.

### Dissemination

After study completion, the results of the primary and secondary analyses will be published in international peer-reviewed journals (at least one specialized in addiction and another in adolescent psychiatry).

If shown to provide valid and reliable information in addition to traditional measures, findings would be presented in international symposiums to consider ways to develop new research protocols on dual disorders in adolescents, which involved these applications.

Findings regarding the usefulness and possible benefits of the Stop-Cannabis app will be transmitted to the team from the University of Geneva who programed the app to suggest possible areas for improvement.

## Author Contributions

XB, YE, DC, and YK: substantial contributions to the conception and design of the article; XB, YE, AC, JB, J-FE, DC, and YK: drafting the article or revising it critically for important intellectual content, final approval of the version to be published, and agreement to be accountable for all aspects of the article in ensuring that questions related to the accuracy or integrity of any part of the article are appropriately investigated and resolved.

## Conflict of Interest Statement

The research was conducted in the absence of any commercial or financial relationships that could be construed as a potential conflict of interest. YK was involved in the development of Stop-Cannabis website and app.

## Footnotes

^1^https://itunes.apple.com/ch/app/stop-cannabis/id532498307?l=fr&mt=8

^2^http://www.stop-cannabis.ch

^3^https://oraprdnt.uqtr.uquebec.ca/pls/public/docs/GSC3472/F463443489_DEP_ADO_ang_V3_2.pdf
